# Promoter methylation and downregulation of *SLC22A18 *are associated with the development and progression of human glioma

**DOI:** 10.1186/1479-5876-9-156

**Published:** 2011-09-21

**Authors:** Sheng-Hua Chu, Dong-Fu Feng, Yan-Bin Ma, Hong Zhang, Zhi-An Zhu, Zhi-Qiang Li, Pu-Cha Jiang

**Affiliations:** 1 Department of Neurosurgery, NO.3 People's Hospital Affiliated to Shanghai Jiao Tong University School of Medicine, Shanghai 201900, China; 2 Department of Neurosurgery, Zhongnan Hospital of Wuhan University, Wuhan 430071, China

## Abstract

**Background:**

Downregulation of the putative tumor suppressor gene *SLC22A18 *has been reported in a number of human cancers. The aim of this study was to investigate the relationship between *SLC22A18 *downregulation, promoter methylation and the development and progression of human glioma.

**Method:**

*SLC22A18 *expression and promoter methylation was examined in human gliomas and the adjacent normal tissues. U251 glioma cells stably overexpressing *SLC22A18 *were generated to investigate the effect of *SLC22A18 *on cell growth and adherence *in vitro *using the methyl thiazole tetrazolium assay. Apoptosis was quantified using flow cytometry and the growth of *SLC22A18 *overexpressing U251 cells was measured in an *in viv*o xenograft model.

**Results:**

*SLC22A18 *protein expression is significantly decreased in human gliomas compared to the adjacent normal brain tissues. *SLC22A18 *protein expression is significantly lower in gliomas which recurred within six months after surgery than gliomas which did not recur within six months. *SLC22A18 *promoter methylation was detected in 50% of the gliomas, but not in the adjacent normal tissues of any patient. SLC22A18 expression was significantly decreased in gliomas with *SLC22A18 *promoter methylation, compared to gliomas without methylation. The *SLC22A18 *promoter is methylated in U251 cells and treatment with the demethylating agent 5-aza-2-deoxycytidine increased *SLC22A18 *expression and reduced cell proliferation. Stable overexpression of *SLC22A18 *inhibited growth and adherence, induced apoptosis *in vitro *and reduced *in vivo *tumor growth of U251 cells.

**Conclusion:**

*SLC22A18 *downregulation via promoter methylation is associated with the development and progression of glioma, suggesting that *SLC22A18 *is an important tumor suppressor in glioma.

## Background

Gliomas are a major class of human intrinsic brain tumors, which includes well differentiated low grade astrocytomas, anaplastic astrocytomas and glioblastoma multiforme, the most malignant brain tumor of adulthood. Although resection remains the most effective treatment for glioma, the high rate of postoperative recurrence inevitably leads to a poor clinical outcome. In an effort to develop novel potential effective treatments, recent studies have focused on understanding the molecular pathogenesis of glioma formation and progression. Gliomas are frequently characterized by invasion and growth [1] and are hypothesized to form in a multistage process resulting from the accumulation of genetic changes, including p53 and PTEN inactivation [2] and activation of hypoxia-inducible factor 1α, VEGF [3] and c-Met [4].

Solute carrier family 22 (organic cation transporter) member 18 (*SLC22A18*), also known as *IMPT1/BWR1A/TSSC5*, is located within the human 11p15.5 cluster [5,6]. Blast homology analysis suggests that *SLC22A18 *is a member of the family of polyspecific transporters and multidrug resistance genes [6]. More recently, SLC22A18 has been shown to be a tumor suppressor candidate and a substrate for RING105 [7]. Structural mutations in SLC22A18 are rare, with isolated reports of point mutations in a breast cancer cell line, a rhabdomyosarcoma cell line [6], and Wilms' tumors and lung tumors [5]. Exonic deletions in Wilms' tumors [5] and loss of heterozygosity in hepatoblastomas [8] have also been reported, indicating that SLC22A18 may play a role in tumorigenesis. In the current study, we sought to determine the functional role of SLC22A18 in gliomas, in order to define the relationship between *SLC22A18*, promoter methylation and tumor behavior.

## Methods

### Patients and specimens

Sixty surgically resected human glioma specimens and the corresponding adjacent normal brain tissues were collected at the Department of Neurosurgery, Zhongnan Hospital of Wuhan University between 2004 and 2005 and the NO.3 People's Hospital Affiliated to Shanghai Jiao Tong University School of Medicine between 2006 and 2008. Informed patient consent and prior approval from the Zhongnan Hospital of Wuhan University and NO.3 People's Hospital Affiliated to Shanghai Jiao Tong University School of Medicine Ethics Committees (Ethic approval ZNHWHU0387, NTPHSHJTUSM045) was obtained before the clinical materials were used for research purposes. All experiments on humans in the present study were performed in compliance with the Helsinki Declaration. All tumor specimens were pathologically confirmed as glioma. Thirty specimens diagnosed as low grade (WHO I-II) glioma and 30 diagnosed as high grade (WHO III-IV) glioma were chosen for comparison. Of the 60 patients, 45 patients were male and 15 were female, with an age range of 28-58 years (average 43.4 years). All specimens were stored at -80°C until analysis.

### Immunostaining

Adult mouse brain was frozen in Tissuetek, and 6-10 μm sections were cut using a cryostat. The sections were fixed in methanol at -20°C for 10 min, washed and blocked with 0.1% BSA in PBS and the sections were sequentially incubated with primary antibody (1 hr), the biotinylated secondary avidin-biotin complex (Vector Laboratories, Burlingame, CA, USA) and diaminobenzidine substrate (Sigma, St. Louis, MO, USA). After washing, the sections were counterstained with methyl green and mounted in Permount (Fluka, Buchs, Switzerland).

For immunostaining of cultured cells, cells were fixed for 10 min in methanol at -20°C, washed with PBS, blocked with 0.1% BSA, stained with primary antibody followed by a rhodamine- or fluorescein-conjugated secondary antibody and mounted in Vectashield (Vector Laboratories). The antibodies used were SMI312 (Sternberger Monoclonals, Baltimore, MD, USA) which is a cocktail of monoclonal antibodies directed against phosphorylated epitopes on the M and H neurofilament (NF) subunits Anti-*β*-tubulin III monoclonal antibody (Sigma), anti-HA tag monoclonal antibody 16B12 (Babco, Richmond, CA, USA), anti-GalC and anti-GFAP monoclonal antibodies (Boehringer Mannheim GmbH, Mannheim, Germany), anti-p27^Kip1 ^monoclonal (Santa Cruz Biotechnology, Santa Cruz, CA, USA) and anti-*β*-tubulin monoclonal antibodies (Sigma and Boehringer Mannheim) which gave similar results. The secondary antibodies were obtained from Jackson ImmunoResearch (West Grove, PA, USA).

### Cell culture and cell growth

U251 cells (Wuhan University of China), human astrocytes, oligodendrocytes and neurons (ScienCell Research Laboratories, San Diego, CA, USA) were cultured in RPMI-1640 (Gibco Life Technologies, Paisley, Scotland, UK) supplemented with 10% fetal bovine serum and penicillinstreptomycin. To establish U251 cell growth curves, 1 × 10^4 ^cells were seeded in 6 cm diameter plates, and the media was changed every three days. The plates were divided into two groups (n = 9 each) and one was cultured in RPMI-1640, while the other group was cultured in RPMI-1640 containing 2 μM 5-aza-2-deoxycytidine. The cell numbers were counted on days 3, 5 and 7 after seeding. After counting, the cells from each group were subject to Western blotting. The optimal concentration of the demethylation agent had previously been determined by culturing U251 cells in 0, 1, 2, 4, 8, 10, 20 and 40 μmol/ml 5-aza-2-deoxycytidine and 2 μM was chosen as the highest concentration at which cells survived without drug-related cell death observed in the culture.

### Construction of the *SLC22A18 *expression vector

The 890 base pair *SLC22A18 *cDNA was amplified using reverse transcription polymerase chain reaction (RT-PCR) [9]. The PCR reaction (10 μl) contained 1 μl cDNA, l μl 10 × buffer (MgCl_2_), 0.4 mM dNTPs, 1 umol primer, 1 U TaqDNA Polymerase. After denaturation at 95°C for 5 min, PCR was performed for 35 cycles (30 s at 95°C, 30 s at 50°C and 30 s at 72°C) and extended at 72°C for 5 min. The linear NHeI-EcoRI fragment containing the SLC22A18 cDNA was subcloned into pcDNA3.1 (Invitrogen Company), which yielded pcDNA3.1-SLC22A18 by T4 ligase (TaKaRa Company). The insertion of SLC22A18 in pcDNA3.1 was confirmed by PCR, restriction enzyme digestion analysis (NHeI and EcoRI) and DNA sequencing.

### Determination of the optimal concentration of G418

G418 is an aminoglycoside which is commonly used as a selective agent for the bacterial neoR/kanR genes. U251 cells were incubated at 37°C in RPMI-1640 medium supplemented with 10% calf serum, 100 u/ml penicillin and 100 μg/ml streptomycin, in an atmosphere of 5% CO_2 _at saturated humidity. The culture medium was changed every 48 h. The optimal concentration of G418 was determined by plating U251 cells at 5 × 10^4 ^per well in 2 ml media in 24 well plates G418 (Sigma) was added at 50, 100, 150, 200, 300, 400, 500, 600, 700 or 800 μg/ml and the culture media were changed every 48 h. The lowest G418 concentration, at which all cells died after 12-14 days culture was chosen as the optimal concentration for selection of resistance.

### Transfection of U251 cells with pcDNA3.1-SLC22A18 and PCR confirmation of *SLC22A18 *expression in U251 cells

To stably transfect the SLC22A18 gene, 1 × 10^6 ^U251 cells were plated in 6 well plates 24 h before transfection and lipofectamine 2000 (Invitrogen Company) was used to transfect 5.0 μg of pcDNA3.1-SLC22A18 or 5.0 μg of empty pcDNA3.1 according to the manufacture's instructions. After 48 h, the transfected cells were selected in media supplemented containing 150 μg/ml G418 and the media was changed every 48 h. Non-transfected U251 cells died within two weeks. G418-resistant pcDNA3.1-SLC22A18 transfected cells were named U251-SLC22A18 and G418-resistant cells transfected pcDNA3.1 were named as U251-EV.

To confirm the stable transfection of SLC22A18, DNA from untransfected U251 cells, U251-EV and U251-SLC22A18 cells was isolated using the Puregene™ DNA Isolation Kit (Gentra systems, Minneapolis, MN, USA). Primers were designed against *SLC22A18 *[10], forward (5'- AGCTGAGCAGCCACTTCTC -3') and reverse (5'- AAAGCTGCGGTACAGGAGG -3') and PCR was performed in 50 μl reactions containing 3 μl cDNA, 5 μl 10 × Buffer, 4 μl MgC12, 1 μl dNTPs, 1 μl primers and 0.3 μl *Taq *DNA Polymerase with an initial denaturation step of 94°C for 7 min, followed by 30 cycles of a three-step program of 94°C for 30 s, 56°C for 30 s and 72°C for 45 s, followed by a final extension step at 72°C for 7 min. The PCR products were electrophoresed on an agarose gel.

### Measurement of cell growth

Cell proliferation was measured using the methyl thiazole tetrazolium (MTT) assay [11]. Cells were seeded in 24-well plates at a density of 1 × 10^4 ^cells/well and 24 hr later 200 μl 5 mg/μl MTT (Sigma) in PBS was added to each well incubated for 4 h at 37°C and the precipitate was solubilized in 100 μl 100% dimethylsulfoxide (Sigma) with shaking for 15 min. Absorbance values were determined using an enzyme-linked immunosorbent assay reader (Model 318, Shanghai, China) at 540 nm. Each assay was performed nine times and the results are expressed as the mean ± SE compared to the control.

### Measurement of apoptosis by flow cytometry

U251 cells were centrifuged 72 hr after transfection after transfection washed with PBS fixed in 7% cold ethanol treated with 10 g/L RNase resuspended and stained with 10 g/L propidium iodine. After 30 min at room temperature in the dark, the cells were analyzed by scanning flow cytometer to determine the percentage of apoptotic cells, defined as cells in the cell cycle distribution with a lower DNA content than G_1 _cells.

### Adhesion assay

Cells were seeded in quadruplicate at a density of 1 × 10^4 ^cells/well in 96 well plates coated with 10 g/L BSA, 50 mg/L Matrigel, or 10 mg/L fibronectin (Fn), cultured at 37°C for 60 min, and the MTT assay was performed as previously described [12-15]. Each assay was performed in triplicate.

### Tumor cell adherence to ECV304

ECV304 cells were plated in 96 well plates at a density of 5 × 10^4 ^cells/well cultured for 48 hr, the supernatant was aspirated and U251, U251-EV or U251-SLC22A18 cells were plated at a density of 5 × 10^4 ^cells/well and cultured for 30 min. The wells were washed twice with PBS to remove unattached cells 100 μl 25% rose Bengal solution was added, incubated for 5 min, the supernatant was aspirated, the wells were washed twice with PBS. 200 μl 95% ethanol/PBS (1:1) was added, incubated for 20 min and absorbance was measured at 540 nm. Each assay was performed in triplicate.

### Murine xenograft model

Male 4 to 6 week old BALB/c athymic nude mice were subcutaneously injected with 2 × 10^6 ^U251, U251-EV or U251-SLC22A18 cells. Tumor diameters were measured at regular intervals with digital calipers, and the tumor volume in mm^3 ^was calculated using the formula: volume = (width)^2 ^× length/2. The animal experiments in this study were performed in compliance with the guidelines of the Institute for Medical School Institutes at Wuhan University and Shanghai Jiao Tong University.

### Immunohistochemical staining

The excised tumors were paraffin-embedded and 4 μM sections were prepared. Antigen retrieval was performed by boiling in citrate buffer for 15 min and peroxidase activity was blocked using 0.3% peroxide in absolute methanol. The sections were incubated with anti-SLC22A18 polyclonal antibody (1:100; Sigma) at 4°C overnight, washed twice with PBS and incubated with secondary antibody (Santa Cruz, CA) at room temperature for 30 min. After washing, the sections were incubated with immunoglobulins conjugated with horseradish peroxidase, staining was visualized using 3, 3'-diaminobenzidine and the sections were calculated from the total number of cells observed in least 10 randomly chosen non-overlapping high-power (×400) fields in each case. SLC22A18 expression was graded on a scale from + to +++, with 0 to ≤25% positive cells graded +; > 25% to < 50% graded ++ and > 50% graded +++.

### DNA extraction and MSP

Briefly, genomic DNA was extracted from tumor tissues, the corresponding normal brain tissues and the U251 cells by the digestion with proteinase K using the Genomic DNA Purification Kit (Gentra Systems, Minneapolis, MN, USA) and 1 μg genomic DNA was treated with the Chemicon CpG WIZ™ DNA Modification Kit (Chemicon International, Temecula, CA, USA) to convert unmethylated cytosines to uracil, leaving methylated cytosines unchanged. The modified DNA was diluted in TE buffer. *SLC22A18 *promoter methylation analysis was performed by PCR, using bisulfite-treated DNA as template, with specific primers for the methylated (unmodified by bisulfite treatment) and unmethylated (bisulfite modified) gene sequences using the MSP method [16]. The *SLC22A18 *primer sequences for the unmethylated reaction (UMS sense 5'-CGTTTTTGTAAAGGTAGGTATTCGA-3' and UMAS antisense 5'-AAACTAAAAAAAACAAAACAACCG-3') were designed to amplify a 144 bp product [16]. The *SLC22A18 *primer sequences for the methylated reaction (MS sense 5'-CGTTTTTGTAAAGGTAGGTATTCGA-3' and MAS antisense 5'-AACTAAAAAAAACAAAACAACCACA-3') were designed to amplify a 146 bp product [16]. The results were confirmed by repeating the bisulfite treatment and MSP assays for all samples.

### Total RNA isolation and reverse-transcriptase polymerase chain reaction

Total RNA was extracted from glioma tissues, the adjacent brain tissues, the glioma cell line U251, astrocytes, oligodendrocytes and neurons using TRIzol (Invitrogen, Carlsbad, CA) following the manufacturer's instructions. The RT reaction was performed on 2 μg of total RNA using the SuperScript II First-Strand Synthesis and an oligo(dT) primer (Invitrogen). The *SLC22A18 *primer sequences and RT-PCR conditions were as previously described (forward primer 5'-GCTTCGGCGTCGGAGTCAT-3' and reverse primer 5'-AGCCTGGGCGTCAGTTTT-3') [16]. The housekeeping gene *GAPDH *was used as an internal control for the RT reaction (forward primer 5'-GGGAGCCAAAAGGGTCATCATCTC-3' and reverse primer 5'-CCATGCCAGTGAGCTTCCCGTTC-3') [16]. PCR was performed over 35 cycles at 94°C for 1 min, at 62°C for 1 min, and 72°C for 1 min followed by a final extension at 72°C for 5 min and the PCR products were analyzed using 2% agarose gels.

### Western blotting

Untransfected U251 cells, U251-EV, U251-SLC22A18 cells, 5-aza-2-deoxycytidine-treated cells, astrocytes, oligodendrocytes and neurons were washed in ice-cold PBS and lysed in buffer using standard methods [17]. Brain tissues were homogenized using a homogenizer in RIPA lysis buffer (50 mM Tris-HCl, pH 8.0, 1% NP40, 0.1% SDS, 100 μg/ml phenylmethylsulfonyl fluoride, 0.5% sodium deoxycholate, 0.02% sodium azide, 1 μg/ml aprotinin and 150 Mm NaCl) on ice. The supernatants were collected after centrifugation at 14,000 × g at 4°C for 10 min, protein concentration was determined and whole-tissue lysates were mixed with an equal amount 5X SDS loading buffer (125 mM Tris-HCl, 4% SDS, 20% glycerol, 100 mM DTT and 0.2% bromophenol blue) as previously described [18]. Samples were heated at 100°C for 5-10 min and were separated on pre-cast 10% SDS-polyacrylamide gels (Fluka, Ronkonkoma, NY, USA), electrotransferred onto nitrocellulose membranes (Invitrogen) blocked for 1 h at room temperature in 5% non-fat milk in TBS (20 mM Tris-HCl, 150 mM NaCl and 0.1% Tween-20), incubated overnight at 4°C with anti-SLC22A18 antibody (1:1,000), then with a horseradish peroxidase-conjugated secondary antibody (1:5,000) for 1 h at room temperature in 1% non-fat milk in TBS and the bands were visualized using the enhanced chemiluminescence system (Pierce, Rockford, IL, USA).

### Data analysis

Statistical differences between groups were examined using the Fisher's exact test. *P*-values less than 0.05 were considered statistically significant.

## Results

### SLC22A18 protein expression is downregulated in glioma

**Figure 1:**
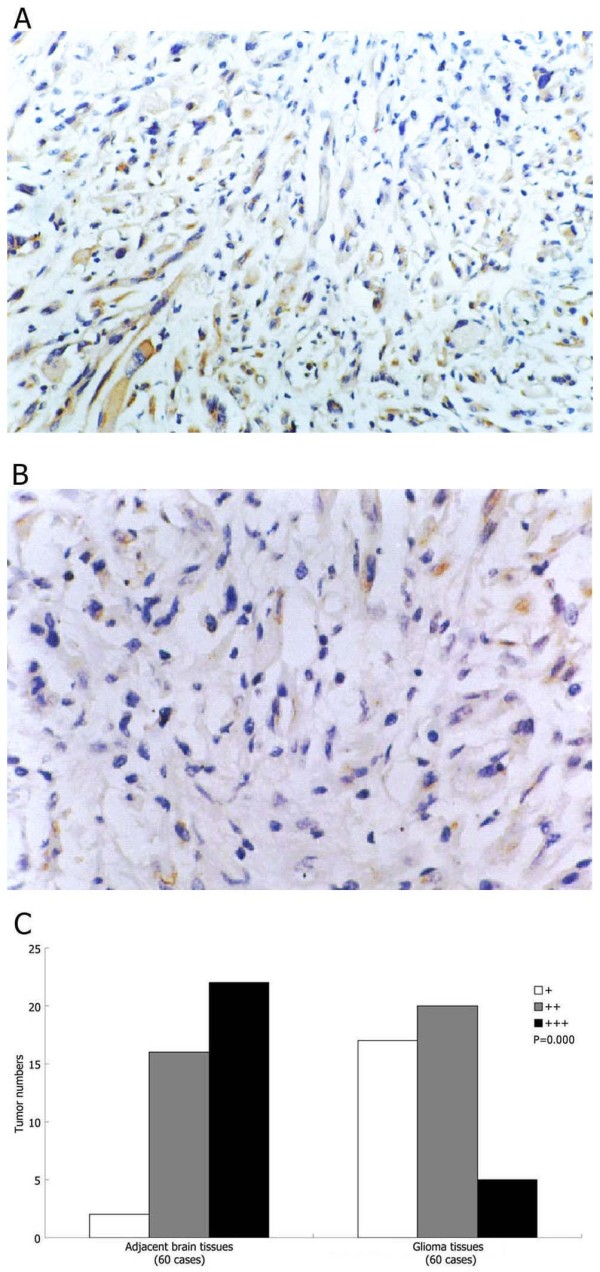
***SLC22A18 *is downregulated in glioma**. Representative images of *SLC22A18 *immunohistochemical staining in (A) adjacent normal brain tissue and (B) glioma, × 200. (C) Semiquantitative analysis of *SLC22A18 *expression in glioma and the adjacent brain tissue, *P *value compares overall *SLC22A18 *expression in each group.

We examined the expression of *SLC22A18 *gene in 60 gliomas and the adjacent normal brain tissues using immunohistochemistry. In the adjacent normal brain tissue, brown positive staining was mostly homogeneously distributed within the cytoplasm (Figure 1A). In glioma tissues, *SLC22A18 *was expressed at reduced levels compared to normal tissue (Figure 1B). Semi-quantitative analysis indicated a significant reduction in *SLC22A18 *expression in gliomas and the corresponding adjacent brain tissue (*P *= 0.000, Figure 1C).

### Downregulation of *SLC22A18 *protein expression is associated with malignancy in glioma

**Figure 2:**
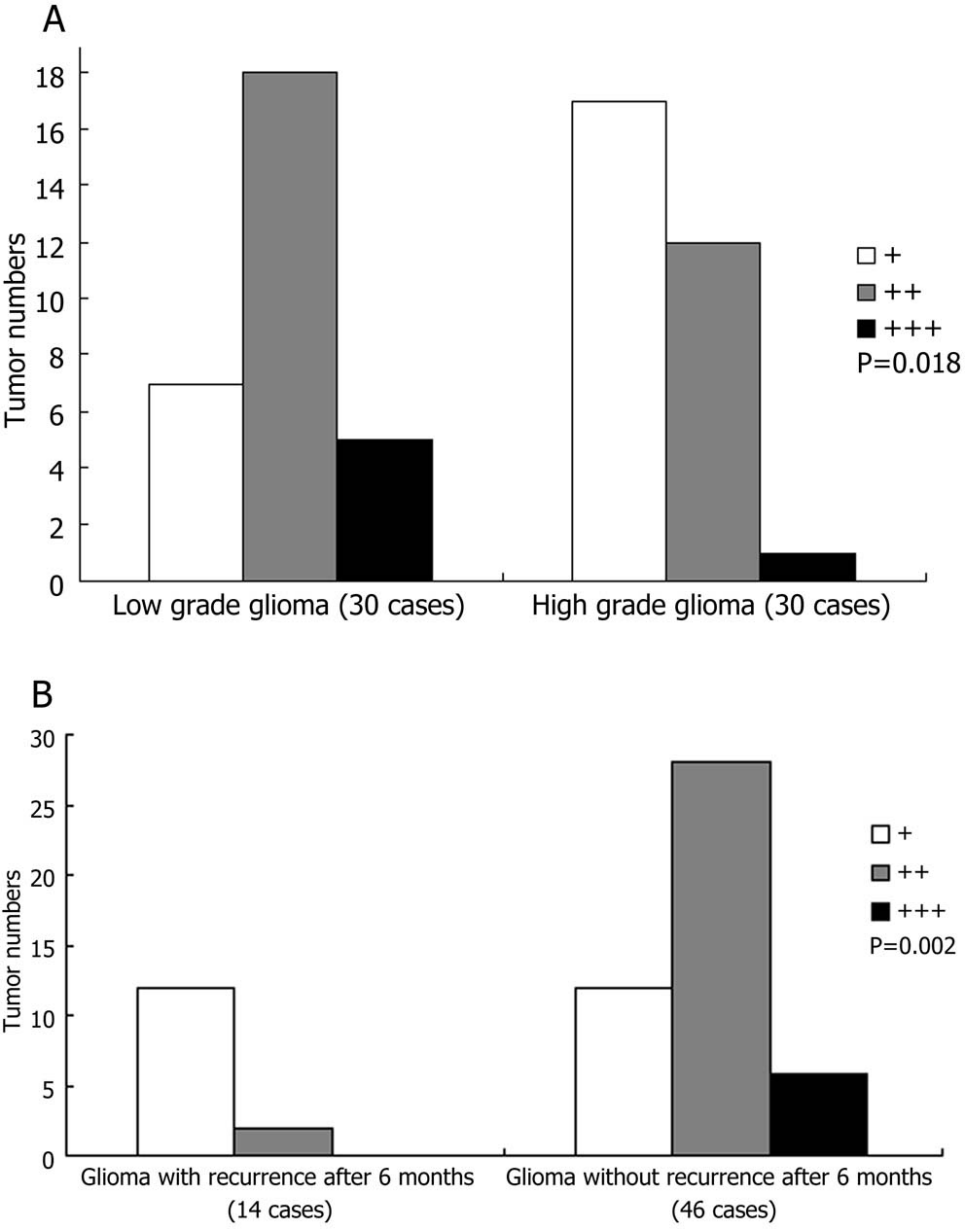
***SLC22A18 *expression in patients stratified by tumor grade and recurrence**. (A) Semiquantitative analysis of *SLC22A18 *immunohistochemical staining in low grade (WHO I-II) glioma and high grade (WHO III-IV) glioma. (B) Semiquantitative analysis of *SLC22A18 *immunohistochemical staining in gliomas which recurred and those which did not recur within six months after surgery, *P *values compare overall *SLC22A18 *expression in each group.

To evaluate the correlation between the *SLC22A18 *expression and the biological characteristics of glioma, SLC22A18 protein expression was compared in 30 patients with high grade (WHO III-IV) glioma and another 30 patients with low grade (WHO I-II) gliomas. *SLC22A18 *expression was significantly lower in the high grade gliomas than low grade gliomas (*P *= 0.018, Figure 2A). *SLC22A18 *expression in the 14 glioma specimens obtained from patients with recurrences six months after surgery was significantly lower than in the 46 specimens from patients without recurrences six months after surgery (*P *= 0.002, Figure 2B).

### Aberrant promoter methylation contributes to *SLC22A18 *downregulation

**Figure 3:**
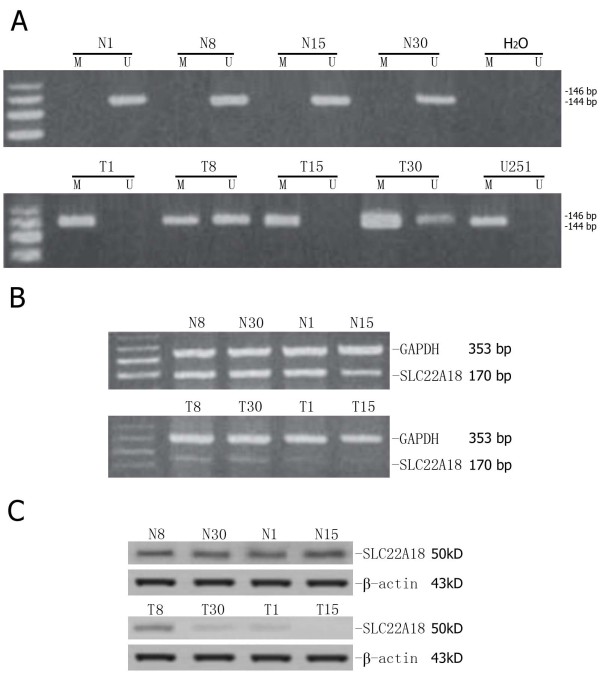
**Correlation between *SLC22A18 *promoter methylation and *SLC22A18 *mRNA and protein expression**. (A) *SLC22A18 *promoter methylation analysis. In patients 1, 8, 15 and 30, the *SLC22A18 *promoter was methylated in glioma and not the adjacent brain tissue. The *SLC22A18 *promoter is also methylated in U251 cells. T, glioma; N, adjacent brain tissue; m, methylated; u, unmethylated. (B) *SLC22A18 *RT-PCR mRNA expression in patients 1, 8, 15 and 30. GAPDH was used as an internal control. (C) Western blot of *SLC22A18 *protein expression in patients 1, 8, 15 and 30. ß-actin was used as an internal control. Both *SLC22A18 *mRNA and protein expression are significantly downregulated in gliomas with promoter methylation, compared to the corresponding adjacent normal brain tissues.

**Figure 4:**
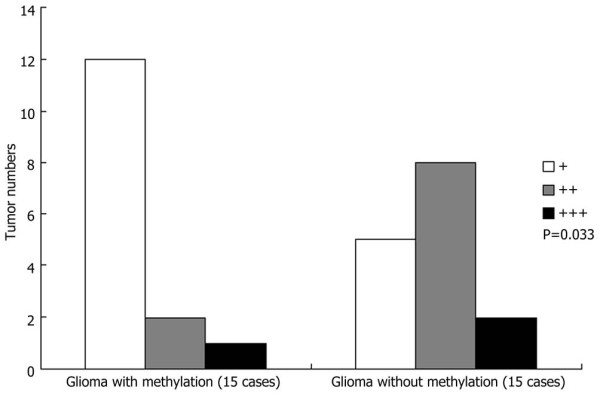
**Semiquantitative analysis of *SLC22A18 *protein expression in gliomas with and without *SLC22A18 *promoter methylation**. P-value compares overall *SLC22A18 *expression in each group.

To explore whether aberrant promoter methylation was responsible for the downregulation of *SLC22A18 *in glioma tissues, the methylation status of the *SLC22A18 *promoter and *SLC22A18 *expression were correlated in the 30 glioma specimens and the corresponding normal tissues. Promoter methylation occurred in gliomas from 15/30 patients and was absent in all of the adjacent brain tissues (Figure 3A). The *SLC22A18 *methylation status and clinicopathological characteristics of all 30 glioma patients are shown in Table 1. RT-PCR analysis indicated that *SLC22A18 *mRNA was significantly decreased or absent in all of the 15 gliomas in which the *SLC22A18 *promoter was methylated, compared to adjacent normal brain tissues (Figure 3B). Furthermore, Western blotting analysis demonstrated that in the 15/30 glioma samples with *SLC22A18 *promoter methylation, SLC22A18 protein expression was significantly decreased compared to the adjacent normal brain tissue (Figure 3C). Semiquantitative analysis of immunohistochemical staining indicated that *SLC22A18 *expression in the 15 glioma samples with promoter methylation was significantly lower than the other 15 glioma samples without promoter methylation (*P *= 0.033, Figure 4). This findings suggesting that promoter methylation contributes to *SLC22A18 *regulation in gliomas. Furthermore, of the 15 patients with glioma *SLC22A18 *promoter methylation, 10/15 recurred within six months after surgery, indicating that *SLC22A18 *promoter methylation and protein downregulation is associated with glioma recurrence. However, compared to normal tissues, SLC22A18 mRNA and protein expression were downregulated in 26 of the 30 glioma samples tested, yet *SLC22A18 *promoter methylation was only observed in 15/30 of these gliomas. This data demonstrates that promoter methylation is involved in the downregulation of *SLC22A18 *in gliomas, but that other mechanisms also regulate *SLC22A18 *expression.

**Table 1:** *SLC22A18 *methylation status and clinicopathological findings in 30 patients with glioma

**Patient no**.	Age (yr)	Sex	Tumor size(cm)	Recurrence within six months	Pathological grade	Methylation status	*SLC22A18 *protein expression rate (%)	
							
							Glioma	Adjacent brain tissue
1	53	M	3.5 × 4 × 3	+	IV	+	4	33
3	42	M	3 × 4.5 × 5	+	III	+	25	58
8	38	F	3.5 × 3 × 3	+	III	+	35	62
9	49	M	3 × 4 × 3	+	IV	+	3	42
10	58	M	5.5 × 4 × 4	-	II	+	39	67
15	69	M	3.5 × 4 × 3	+	IV	+	8	38
17	32	M	5 × 6 × 4	-	II	+	37	65
19	45	F	4 × 5 × 6	-	II	+	34	59
20	51	M	4.5 × 5 × 6	+	IV	+	0	25
21	43	M	3.5 × 4 × 5	+	IV	+	9	35
24	48	M	3.5 × 4 × 4	-	II	+	32	61
25	55	F	3.5 × 4 × 5	-	II	+	30	59
27	62	F	4 × 4 × 3	+	IV	+	0	30
29	34	M	5 × 4 × 3	-	II	+	37	65
30	39	M	3.5 × 2 × 3	+	IV	+	6	38
2	71	M	2 × 3 × 4	-	I	-	45	68
4	33	M	3 × 4 × 5	-	II	-	32	61
5	43	M	3 × 4 × 6	-	I	-	46	62
6	46	F	3.5 × 4 × 4	+	III	-	25	57
7	48	M	4.5 × 4 × 5	-	II	-	37	68
11	47	F	5.5 × 5 × 6	+	IV	-	12	54
12	57	M	5 × 6 × 5	-	II	-	32	70
13	56	M	3.5 × 5 × 6	-	I	-	52	75
14	46	M	3.5 × 5 × 4	-	I	-	45	72
16	57	M	3 × 3 × 4	+	IV	-	14	62
18	53	M	3 × 4 × 5	-	I	-	52	78
22	54	F	5.5 × 6 × 5	-	I	-	48	73
23	65	M	3 × 4 × 3	+	III	-	34	65
26	46	F	3.5 × 5 × 4	-	I	-	52	82
28	62	M	3 × 4.5 × 5	+	III	-	29	62

### Promoter demethylation increases *SLC22A18 *expression and reduces U251 cell growth

**Figure 5:**
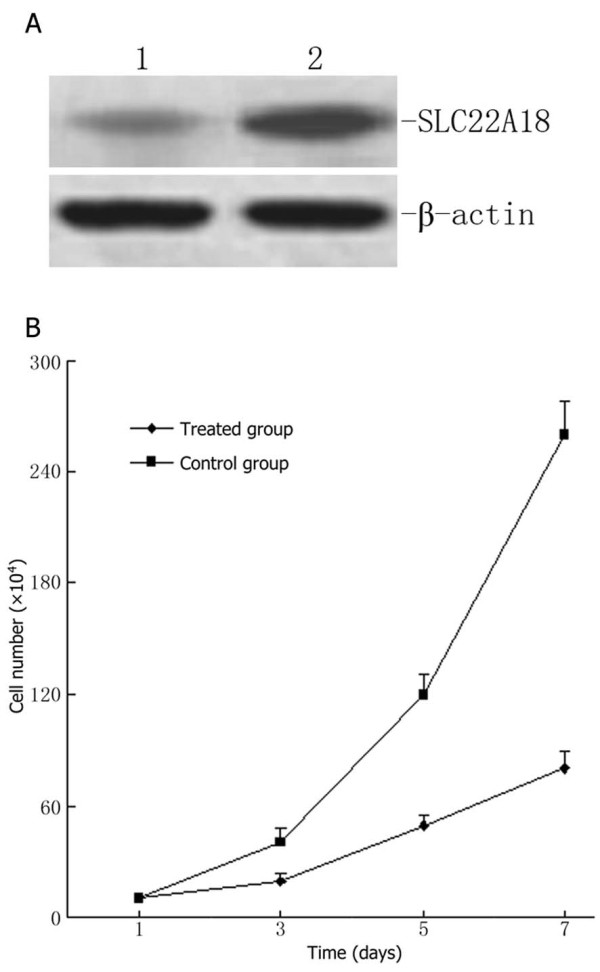
**The demethylating agent 5-aza-2-deoxycytidine restores *SLC22A18 *expression in U251 cells and suppresses cell growth**. (A) Western blotting indicating a significant increase in *SLC22A18 *expression after treatment of U251 cells with 2 μg/ml 5-aza-2-deoxycytidine for 7 days: 1, control cells; 2, treated cells; ß-actin was used as an internal control. (B) Growth curves demonstrating reduced cell numbers in the 5-aza-2-deoxycytidine-treated group, compared to empty plasmid transfected U251-EV cells and untransfected U251 cells.

We observed that the *SLC22A18 *promoter is methylated in the human glioma cell line U251 (Figure 3A). To study whether demethylation agents can restore SLC22A18 expression, the cells were treated with the demethylation agent 5-aza-2-deoxycytidine (2 μM) for 9 days and the cell number was determined on days 3, 5 and 7. Western blotting demonstrated that SLC22A18 expression in 5-aza-2-deoxycytidine-treated cells increased significantly compared to untreated control cells (Figure 5A). Moreover, increased SLC22A18 expression was associated with a decreased rate of cell growth following 5-aza-2-deoxycytidine treatment (*P *< 0.05, Figure 5B).

### PCR analysis of *SLC22A18 *in U251 cells

**Figure 6:**
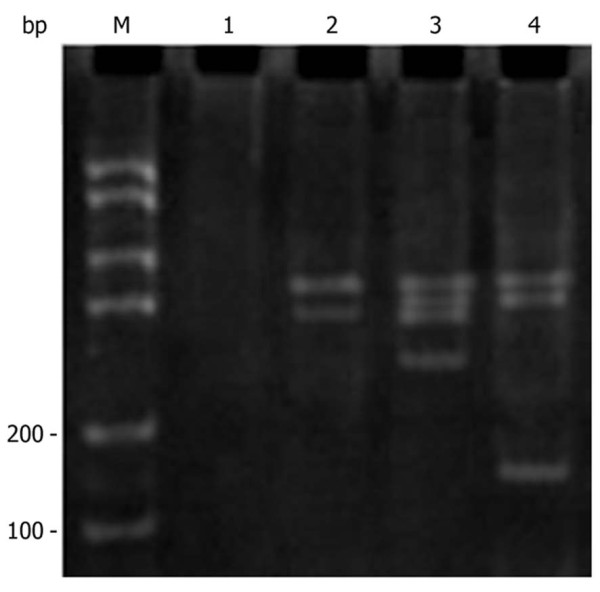
**PCR amplification of *SLC22A18 *in U251-SLC22A18 stable cell lines**. M, DNA molecular mass marker; lane 1, culture medium only control; lane 2, empty plasmid transfected U251-EV cells; lane 3, untransfected U251 cells; lane 4, U251-SLC22A18 cells.

A *SLC22A18 *stable cell line was generated by transfection of U251 cells with *SLC22A18 *cDNA and stable clones were selected using G148. DNA was extracted from untransfected U251, U251-EV and U251-SLC22A18 cells and subjected to PCR using a primer pair designed to amplify. As expected, a 170 bp fragment was observed in U251-SLC22A18 cells, but not in untransfected U251 or empty plasmid transfected U251-EV cells (Figure 6), confirming the stable expression of *SLC22A18 *in U251-SLC22A18 cells.

### SLC22A18 protein is expressed in U251-SLC22A18 cells

**Figure 7:**
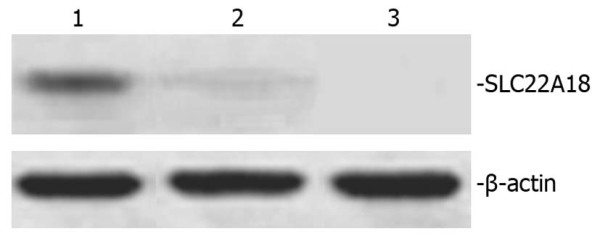
**Western blot of *SLC22A18 *protein expression in U251-SLC22A18 stable cell lines**. Lane 1, U251-SLC22A18 cells; lane 2, empty plasmid transfected U251-EV cells; lane 3, untransfected U251 cells. β-actin was used as loading control.

Total protein was exacted from untransfected U251, U251-EV and U251-SLC22A18 cells and analyzed by Western blotting. A 50 kDa band corresponding to the expected size of SLC22A18 was observed in U251-SLC22A18 cells, but not in U251-EV or U251 cells (Figure 7).

### Cell proliferation assay

**Figure 8:**
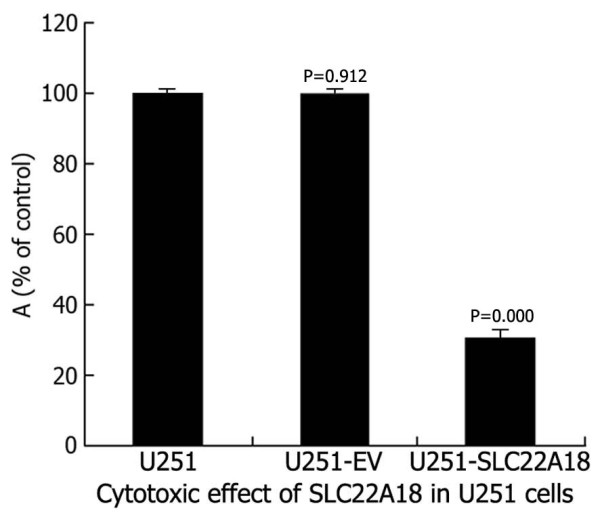
**Cytotoxic effect of *SLC22A18 *expression in U251 cells**. U251-SLC22A18, empty plasmid transfected U251-EV cells and untransfected U251 cells were cultured in plastic 96-well plates and quantified using the MTT assay.

As shown in Figure 8, U251-SLC22A18 caused a statistically significant reduction of cell viability to (31.5 ± 8.56)%, whereas normal U251 and U251-EV had not such change.

### Induction of apoptosis by the SLC22A18 expression

Cells were examined for apoptosis induction by FCM. As shown in Table 2, U251-SLC22A18 induced significant apoptotic response after transfection, about 25.25 ± 4.28 for 24 hours and 30.84 ± 4.72 for 48 hours. However, U251 and U251-EV did not induce any significant apoptotic response until 48 hours after transfection.

**Table 2:** Mean ± SEM apoptotic rate (percentage) in *SLC22A18 *transfected U251 cells

Group (n = 6)	24 h	48 h
Normal U251	1.95 ± 0.32	3.69 ± 0.19
U251-EV	1.95 ± 0.38	3.71 ± 0.21
U251-SLC22A18	25.25 ± 4.28	30.84 ± 4.72

### Effects of SLC22A18 expression on U251 cell adhesion

**Figure 9:**
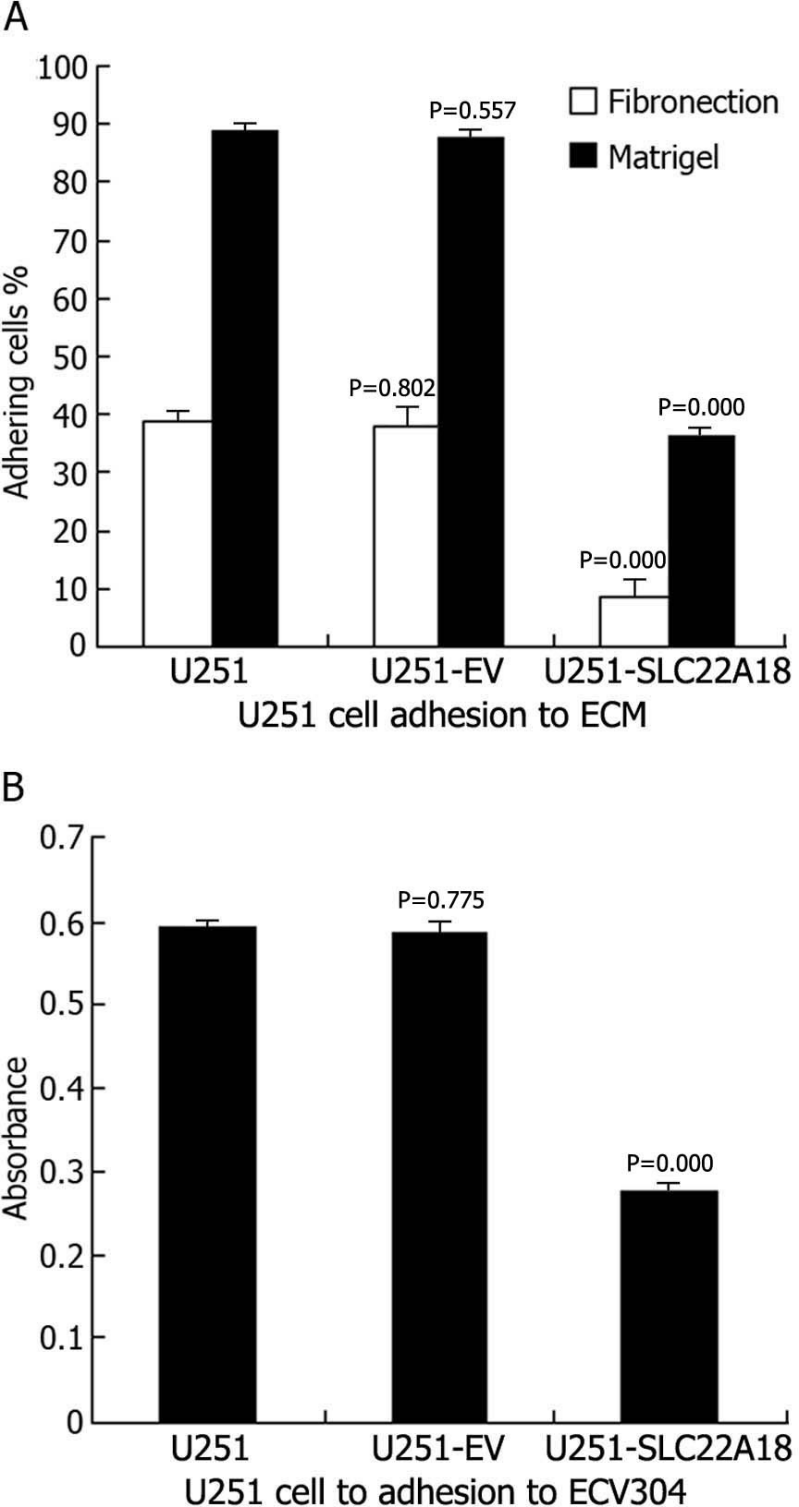
**Effects of *SLC22A18 *expression on U251 cell adhesion**. (A) U251 cell adhesion to ECM (Fn and Matrigel). (B) U251 cell adhesion to ECV304.

Upregulated *SLC22A18 *expression had a clear inhibitory effect on the adhesion of transfected U251 cells to the extracellular matrix (ECM) [Matrigel and Fn] and to ECV304. The percentages of adhesion to ECM were as follows: U251, (39.2 ± 2.21)% (Fn) and (89.4 ± 1.52)% (Matrigel); U251-EV, (38.6 ± 3.18)% (Fn) and (88.6 ± 1.54)% (Matrigel); U251-SLC22A18, (8.5 ± 3.16)% (Fn) and (38.2 ± 1.57)% (Matrigel) (Figure 9A). The tumor cell lines showed different absorbance abilities: U251, 0.592 ± 0.008; U251-EV, 0.589 ± 0.015; U251-SLC22A18, 0.264 ± 0.012 (Figure 9B). Thus, the adhesion of U251-SLC22A18 to ECM and to ECV304 cells was significantly suppressed.

### Effects of *SLC22A18 *expression on tumor growth *in vivo*

**Figure 10:**
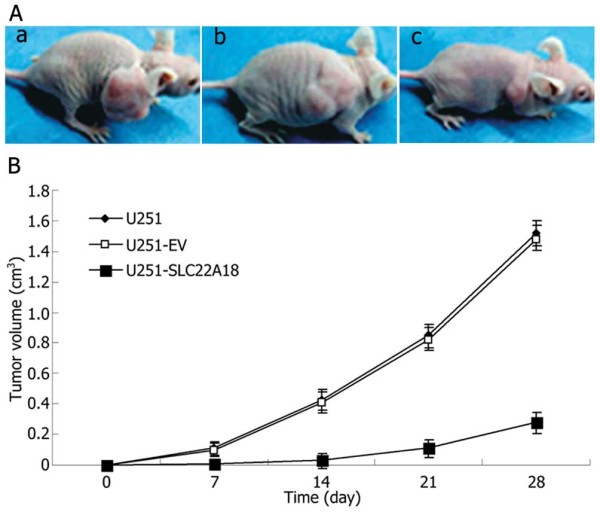
**Effects of *SLC22A18 *expression on tumor growth in vivo**. (A) Subcutaneous tumor model. a, U251 cells group; b, U251-EV cells group; c, U251-SLC22A18 cells group. (B) Tumor growth curves of each group over 28 days.

As shown in Figure 10, U251 and U251-EV xenograft tumors formed and grew rapidly. In contrast, U251-SLC22A18 xenograft tumor formation was significantly delayed. At the end of the experiment, the U251-SLC22A18 tumors were significantly smaller than the tumors from untransfected U251 and U251-EV negative control cells.

### Expression of *SLC22A18 *in normal neurons, astrocytes and oligodendrocytes

**Figure 11:**
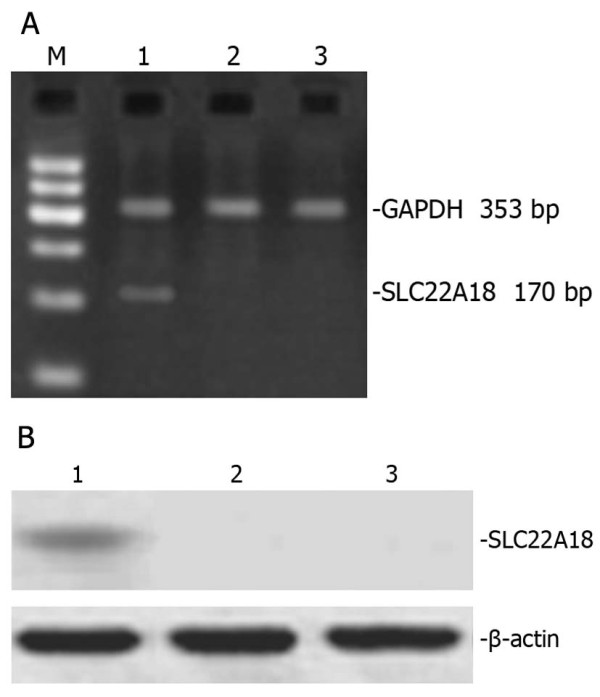
***SLC22A18 *mRNA and protein is expressed in neurons but not astrocytes or oligodendrocytes *in vitro***. *SLC22A18 *RT-PCR (A) and Western blot (B). Lane 1, neurons; lane 2, astrocytes; lane 3, oligodendrocytes; M, standard DNA molecular mass marker.

We analyzed the expression of *SLC22A18 *mRNA and protein in normal neurons, astrocytes and oligodendrocytes. Neurons expressed both *SLC22A18 *mRNA and protein, while astrocytes and oligodendrocytes did not express either *SLC22A18 *mRNA or protein (Figure 11).

### Expression of *SLC22A18 *in adult mouse brain

**Figure 12:**
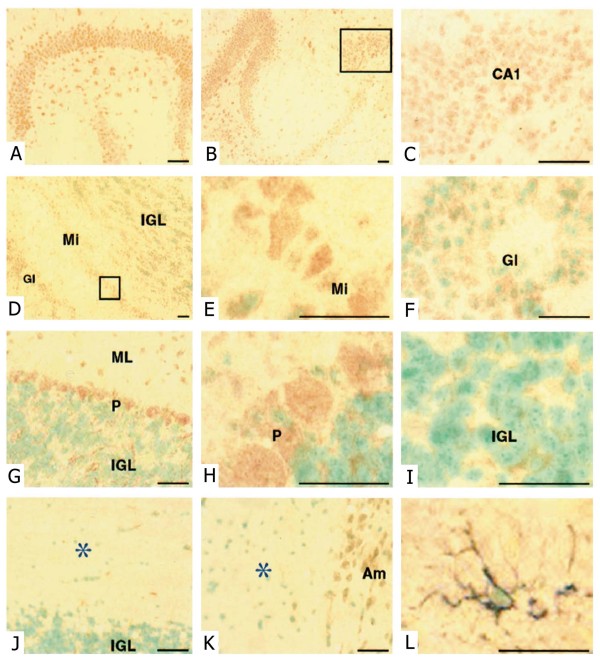
***SLC22A18 *mRNA and protein is expressed in neurons but not astrocytes or oligodendrocytes *in vivo***. (A, B) *SLC22A18 *immunohistochemical staining in the hippocampus and (C) at high magnification in CA1. (D) Olfactory bulb including glomeruli (Gl), mitral cells (Mi), and the inner granule layer (IGL). (E) High magnification view of mitral cells. (F) Small *SLC22A18*-positive periglomerular cells. (G) Cerebellum showing weak staining of the internal granule layer (IGL), strong staining of Purkinje cells (P) and occasional positive cells in the molecular layer (ML). No staining of dendrites was evident in the ML. (H) High magnification of Purkinje cells. (I) High magnification of the IGL, showing cytoplasmic *SLC22A18 *staining. (J) White matter tracts in the cerebellum indicated by *. (K) Corpus callosum indicated by * with *SLC22A18*-positive amygdala (Am) observed at the right edge. (L) High magnification of a double stained astrocytic cell (*SLC22A18*, red brown; GFAP, blue gray) in white matter tracts of the cerebellum. All sections were counterstained with methyl green, scale bars = 20 μm.

To identify the cells which express SLC22A18, we performed SLC22A18 immunostaining on sections of adult mouse brain. Strong SLC22A18 immunostaining was observed in the cortex and cerebellum, but little reactivity was detected in the brainstem. Representative examples of SLC22A18 immunostaining are shown in Figure 12. Hippocampal neurons in all regions (Figure 12A-C) showed strong staining. In the olfactory bulb (Figure 12D-F), mitral cells demonstrated the strongest immunostaining (Figure 12E). Weaker SLC22A18 staining in periglomerular cells (Figure 12F) and very sparse staining in the internal and external plexiform layers was observed. Purkinje cells in the cerebellum (Figure 12G-I) were strongly SLC22A18 positive. The scant cytoplasm of the neurons in the internal granular layer was SLC22A18 positive, while only stellate and basket interneurons were weakly positive in the molecular layer. No staining of dendrites was evident in the molecular layer. SLC22A18 expression was not detected in the white matter tracts of the cerebellum (Figure 12J) even though staining of sequential sections showed the presence of GFAP-positive astrocytes (Figure 12L) and GalC-positive oligodendrocytes (data not shown). Detailed examination of the corpus callosum (Figure 12K) did not reveal SLC22A18 expression in glial cells. Neurons of the amygdala (Figure 12K) were also SLC22A18 positive.

## Discussion

Studies of the underlying molecular mechanisms involved in glioma formation and progression provide tremendous opportunities to identify molecules which may provide novel potential drug design targets for the treatment of brain tumors. Mutations and overexpression of several oncogenes, including c-Met, PDGF and c-myc, have been identified in glioma patients [4,19]. Using molecular analysis, loss of heterozygosity has been observed on several chromosomes in patients with glioma. Many of these chromosomal segments contain known tumor suppressor genes [20,21], such as p16 on 9p, p53 on 17p and RB on 13q. *SLC22A18*, also known as *IMPT1/BWR1A/TSSC5*, is located in the 11p15.5 region and encodes an efflux transporter-like protein with 10 transmembrane domains, whose expression can affect cellular metabolism, cellular growth and drug sensitivity [7]. Human chromosome 11p15.5 is frequently lost in a variety of tumors, including Wilms' tumor, lung cancer, in hepatocarcinoma cells [6] and also in breast cancer, suggesting that one or more tumor suppressor genes map to this region [5]. Exonic deletions of 11p15.5 in Wilms' tumors [5] and loss of heterozygosity in hepatoblastomas [8] have also been reported, indicating that *SLC22A18 *may play a role in tumorigenesis; however, *SLC22A18 *expression in glioma and the relationship with glioma progression has not previously been investigated in humans.

In our study, we demonstrated that *SLC22A18 *protein expression was significantly decreased in gliomas compared to the adjacent brain tissues. We observed that normal neuron express *SLC22A18 *mRNA and protein, but normal astrocytes and oligodendrocytes do not. The lack of detectable *SLC22A18 *expression in human astrocytes and human oligodendrocytes raises the intriguing question of how *SLC22A18 *can influence development of glial tumors. The critical point in developing such a scheme is the identity of the glioma progenitor cell. Although older models proposed that gliomas result from dedifferentiated astrocytes, it recently has been suggested that gliomas are transformed glial precursor cells [22,23]. Recently the discovery that oligodendrocyte precursor cells (OPCs) are the point of origin was reported [24]. Hence, important questions for future studies include whether glial precursor cells *in vitro *and *in vivo *are *SLC22A18 *positive and whether loss of *SLC22A18*, perhaps combined with other genetic lesions, initiates excess cell proliferation. The availability of mouse models will provide answers to these questions as well as an experimental system to develop new therapeutic approaches for gliomas. We also showed that decreased expression of *SLC22A18 *was correlated with an increased post-operative recurrence of glioma, suggesting that downregulation of *SLC22A18 *can affect the degree of malignancy in glioma. Aberrant methylation of the *SLC22A18 *promoter is one major mechanism responsible for the inactivation or downregulation of *SLC22A18 *in a number of tumor types, including breast [6], lung [5] and hepatoblastomas [8]. Our study indicates that aberrant methylation of the *SLC22A18 *promoter occurred in 15 of the 30 gliomas tested, which correlated with significantly reduced *SLC22A18 *mRNA and protein expression suggesting that aberrant promoter methylation contributes to decreased *SLC22A18 *expression in glioma. Of the 15 patients whose gliomas had *SLC22A18 *promoter methylation, 10 recurred within six months after surgery; while only 5 of the 15 without promoter methylation recurred within six months after surgery. Our findings demonstrate that promoter methylation is involved in *SLC22A18 *downregulation in glioma; however, other mechanisms are involved in *SLC22A18 *regulation. It is possible that other epigenetic modifications, such as histone acetylation, may modulate changes in *SLC22A18 *gene expression [25]. Some researchers have reported that dual promoters, P1 and P2, exist for the *SLC22A18 *gene. Sp1 is a transactivator of the P1 promoter, suggesting that DMR may not be associated with the *SLC22A18 *promoters or CpG II islands. The *SLC22A18 *expression was previously reported to be methylation-dependent and histone acetylation-independent [26]. The core *SLC22A18 *promoter lies in the region -120 bp to +78 bp and is devoid of TATA or CAAT boxes. *SLC22A18 *is positively regulated by Sp1 via two functional Sp1 transcription factor binding sites in the promoter region, which are conserved in the human, chimpanzee, mouse and rat gene [27].

To determine whether *SLC22A18 *promoter methylation contributes to increased glioma growth, U251 cells were used to study whether a demethylation agent could restore *SLC22A18 *expression. The demethylation agent 5-aza-2-deoxycytidine restored *SLC22A18 *expression in U251 cells and reduced cell growth. Clearly, as the development of glioma is closely related to both the inactivation of several tumor suppressor genes and overexpression of other genes, overexpression of the single gene *SLC22A18 *could not explain all the biological characteristics of glioma. However, in the present study, we identified that *SLC22A18 *overexpression can effectively inhibit the growth and adhesion of U251 glioma cells, and additionally, overexpression of *SLC22A18 *can trigger apoptosis in glioma cells *in vitro *and delay tumor growth *in vivo*.

## Conclusions

Collectively, our data demonstrates that *SLC22A18 *promoter methylation contributes to the downregulation of *SLC22A18 *in gliomas and reduced *SLC22A18 *expression plays a role in the molecular pathogenesis of glioma, as *SLC22A18 *overexpression can effectively inhibit U251 glioma cell growth and adhesion *in vitro *and tumor growth *in vivo*.

## Abbreviations

*SLC22A18*: solute carrier family 22 (organic cation transporter) member 18; c-Met: hepatocyte growth factor receptor; RPMI: roswell park memorial institute; PBS: phosphate-buffered saline; SDS: sodium dodecyl sulfate.

## Competing interests

The authors declare that they have no competing interests.

## Authors' contributions

SHC, DFF and YBM carried out the laboratory analysis. HZ and ZAZ participated in the design of the study and drafted the manuscript. ZQL and PCJ conceived of the study, and participated in its design and coordination and helped to draft the manuscript. All authors read and approved the final manuscript.
